# Seasonal Migrations of *Pantala flavescens* (Odonata: Libellulidae) in Middle Asia and Understanding of the Migration Model in the Afro-Asian Region Using Stable Isotopes of Hydrogen

**DOI:** 10.3390/insects11120890

**Published:** 2020-12-17

**Authors:** Sergey N. Borisov, Ivan K. Iakovlev, Alexey S. Borisov, Mikhail Yu. Ganin, Alexei V. Tiunov

**Affiliations:** 1 Institute of Systematics and Ecology of Animals, Siberian Branch of the Russian Academy of Sciences, Frunze str., 11, 630091 Novosibirsk, Russia; ivaniakovlev@gmail.com (I.K.I.); baswatch@gmail.com (A.S.B.); 2 All-Russian Research Institute of Brewing, Non-Alcoholic and Wine Industry—A branch of the Gorbatov’s Federal Scientific Center for Food Systems of Russian Academy of Sciences, Rossolimo str., 7, 119021 Moscow, Russia; m-ganin@yandex.ru; 3 A.N. Severtsov Institute of Ecology and Evolution, Russian Academy of Sciences, Leninsky pr. 33, 119071 Moscow, Russia; a_tiunov@mail.ru

**Keywords:** migratory insect, latitudinal migration, dragonfly, globe skimmer, phenology, isotopic ecology, deuterium

## Abstract

**Simple Summary:**

Large-distance migrations of insects have been recognized for many years, but many details of this behaviour remain unknown. The globe skimmer dragonfly has the most extensive cosmopolitan range among all dragonfly species. Migrations of these dragonflies are noted on all continents (except Antarctica), over both land and the oceans, but the patterns of their seasonal movements are still poorly understood. We aimed to confirm seasonal latitudinal migrations of the globe skimmer in Middle Asia and to clarify its migration pattern in extended areas. We used stable isotope composition of hydrogen in wings of dragonflies as an intrinsic marker of their places of origin. Combining phenological data and a comparison with published isotopic data on migratory insects, our results suggest that in spring, the already-mature dragonflies arrive in Middle Asia for reproduction from tropical parts of East Africa and/or the Arabian Peninsula, and, in autumn, summer-generation dragonflies migrate to the south. We conclude that in the Afro-Asian region there is an extensive migration circle of the globe skimmer covering East Africa, Central Asia and the Indian subcontinent with a total length of more than 14,000 km.

**Abstract:**

In Middle Asia, the dragonfly *Pantala flavescens* makes regular seasonal migrations. In spring, sexually mature dragonflies (immigrants) arrive in this region for reproduction. Dragonflies of the aboriginal generation (residents) develop in about two months, and migrate south in autumn. Residents of Middle Asia have significantly lower δ^2^H values (−123.5 (SD 17.2)‰, n = 53) than immigrants (−64.4 (9.7)‰, n = 12), as well as aboriginal dragonfly species from Ethiopia (−47.9 (10.8)‰, n = 4) and the Sahel zone (−50.1 (15.5)‰, n = 11). Phenological data on *P. flavescens* in the Afro-Asian region and a comparison with published isotopic data on migratory insects from this region suggest that (i) the probable area of origin of *P. flavescens* immigrants is located in tropical parts of East Africa and/or the Arabian Peninsula and (ii) the autumn migration of Middle Asian residents to the south may also pass through the Indian Ocean. We assume that in the Afro-Asian region, there is an extensive migration circle of *P. flavescens* covering East Africa, Central Asia and the Indian subcontinent with a total length of more than 14,000 km.

## 1. Introduction

The globe skimmer or wandering glider dragonfly (*Pantala flavescens* Fabricius) is among the most well-known migrants in the insect world. Due to its migration strategy, *P. flavescens* has the most extensive cosmopolitan range among all dragonfly species. This range covers all continents (except Antarctica) [1] and many islands, including the remotest ones, such as Amsterdam Island in the middle of the Indian Ocean [2] and Easter Island in the Pacific Ocean, where *P. flavescens* apparently became non-migratory [3,4]. Migrations of these dragonflies are noted throughout the range, over both land and the oceans [5,6,7,8].

In the tropical part of its range, *P. flavescens* use prevailing seasonal winds related to weather fronts in the intertropical convergence zone. Using these winds, they migrate regularly between Asia and Africa every year [7,9]. Moreover, monsoon rains create ephemeral ponds, which are the primary habitat for larvae of migratory dragonflies [5,8,10].

The migration strategy of *P. flavescens* at the northern limits of its range remains less well known. Our previous research in Middle Asia (southern Kazakhstan, Uzbekistan, Kyrgyzstan, Tajikistan and Turkmenistan) suggested that regular seasonal migrations are characteristic of *P. flavescens* in this region [11]. The northern limit of *P. flavescens* distribution in Middle Asia is 44°50′ N. In spring and early summer, sexually mature dragonflies migrate to Middle Asia. The inflow of immigrants continues from the end of April (probably even earlier in warmer years) until the appearance of dragonflies of the summer generation from June to July. The summer generation develops rapidly over a period of approximately two months. In autumn, dragonflies of the Middle Asian summer generation migrate south, but their destination has not been discovered [11].

Thus, there is little doubt that there are regular seasonal migrations of *P. flavescens* in Middle Asia. However, many aspects of these migrations remain unknown, including the region in which globe skimmers start their spring journey to Middle Asia, the place that their descendants migrate to in autumn, their migration routes and the duration of flights.

One of the main problems in studying insect migrations is how to determine the area of origin of migrants. The small size of insects, in comparison with birds and mammals, limits the use of artificial exogenous marks attached to animals. In this case, the advantage of using naturally occurring endogenous markers, such as stable isotope composition, is obvious [12]. The isotopic composition (or isotopic signatures) of the habitats where the development of an organism took place is reflected and fixed in metabolically inert tissues (for instance, dragonfly wings). This makes it possible to use the global isotope maps of precipitation (isoscapes) to determine the origin of migrants [13,14].

The method of stable isotope analysis is widely used in the study of insect migrations [12,15,16]. This method has been used in the study of dragonflies, including migrations of *Anax junius* (Drury) in North America [14,17] and migrations of *P. flavescens* in northern China [18] and the Maldives [9]. We also used this method in the study of *Sympetrum fonscolombii* (Selys) and proved seasonal latitudinal migrations of this species in Middle Asia [19].

In this work, we conducted stable isotope analysis of hydrogen (D/H ratio, *δ*^2^H) to study migrations of *P. flavescens* in Middle Asia. The main objectives were: (1) to determine the probable region of origin of immigrants that arrive to Middle Asia in spring and (2) to estimate probable migration routes of dragonflies that develop in Middle Asia.

## 2. Materials and Methods

### 2.1. Sample Collection

Sixty-five specimens of *P. flavescens* collected in Middle Asia from 1976 to 2019 (May to October) were used for D/H isotope analysis. Of these, seven specimens of dragonflies collected in southern Kazakhstan were taken from the collection of the Institute of Systematics and Ecology of Animals of the Siberian Branch of the Russian Academy of Sciences (ISEA SB RAS, Novosibirsk). The other 58 specimens (11 exuviae and 47 imagoes) were collected by the first author (S.N.B.) and the third author (A.S.B.). Exuviae were collected in ponds where dragonflies had developed, which guarantees their local origin. This is important, given the high mobility of migratory dragonflies, which are able to migrate large distances after emergence. We used material collected over many years, due to the difficulties in collecting migratory dragonflies in different seasons during one or two field seasons. Along with other authors [14], we assume that dry museum specimens that have been stored for a long time are suitable for isotopic analysis. Adult dragonflies were caught with an entomological net, both near water bodies and at a distance from them, including during autumn migrations in the Eastern Pamirs. In May, the adults were collected in rice paddies where dragonflies congregate for reproduction. Exuviae were collected from paddy fields and other temporary water bodies during the emergence of dragonflies. Identification of exuviae was not difficult, since dragonfly species closely related to *P. flavescens* are absent in Middle Asia.

**Figure 1:**
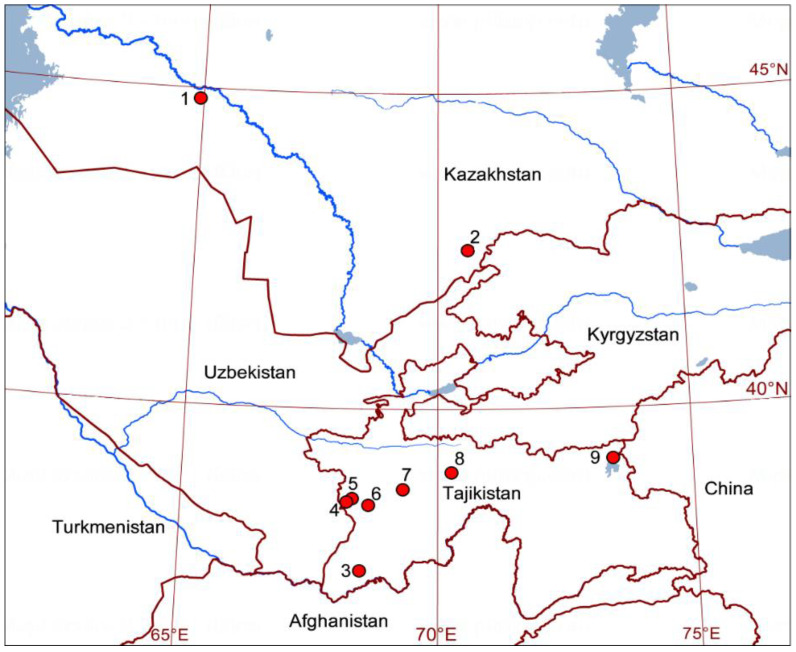
Collection sites of *Pantala flavescens* in Middle Asia. Designations are given in Table 1.

After they were caught, dragonflies were devitalized using ethyl acetate, then dried, and stored on cotton batting. Locations and dates of collection of dragonflies are given in Table 1 and in Figure 1. The *δ*^2^H values of individual samples from Middle Asia are given in Supplementary Table S1 (additional electronic material).

In addition, the wings of four specimens of aboriginal dragonflies (one specimen of *Anax imperator* and three specimens of *Nesciothemis farinosa* from East Africa (Ethiopia, 11°37′ N, 37°25′ E, 1790 m a.s.l., 02–08.VIII.2012, leg. O.E. Kosterin) and 11 specimens of *Anax ephippiger* from West Africa (Mauritania, 20°53′ N, 17°02′ W, 0 m a.s.l., 24.III.1964, collection of ISEA SB RAS)) were analyzed to determine *δ*^2^H values typical of local dragonflies in the hypothesized wintering areas of *P. flavescens*. Dragonflies in Mauritania were collected during spring migrations to the north. Most likely, these specimens developed in the Sahel in winter [20,21,22].

Published data on the δ^2^H value of *Vanessa cardui* butterflies developed in Ethiopia in November [23] and of *P. flavescens* caught in the Maldives [9] were used for comparison.

Maps of mean wind vectors at 850 mb (February to April and September to November, from 1976 to 2020) were obtained using online plotting of the NCEP/NCAR Reanalysis database provided by the National Oceanic and Atmospheric Administration Physical Sciences Laboratory [24].

### 2.2. Stable Isotope Analysis

Dragonfly wings and exuviae were cleaned of lipids in 2:1 chloroform/methanol and dried for three days at 50 °C. Stable hydrogen isotope analysis was conducted using the comparative equilibration method [14,25]. In addition to USGS reference materials, KHS (kudu horn, non-exchangeable δ^2^H_V-SMOW_ = −35.3 ± 1.1‰) and CBS (caribou hoof, non-exchangeable δ^2^H_V-SMOW_ = −157.0 ± 0.9‰), internal laboratory standards were used for equilibration: homogenized wings of three dragonfly species (*Crocothemis erythraea* (Brullé, 1832) from Tajikistan (DS1, δ^2^H = −111.6‰), *Sympetrum sanguineum* (Müller, 1764) from Kazakhstan (DS2, δ^2^H = −110.1‰) and *Sympetrum flaveolum* (Linnaeus, 1758) from Kazakhstan (DS3, δ^2^H = −110.2‰)), fur of mountain hare (*Lepus timidus* Linnaeus, 1758) from the Arctic Circle (Chukotka) (DS4, δ^2^H = −138.2‰) and human hair (DS5, δ^2^H = −70.8‰). The non-exchangeable δ^2^H values for five laboratory standards were obtained from IsoAnalytical Ltd. (Crewe, Great Britain). Non-exchangeable δ^2^H values of laboratory standards were measured using a 3-point linear calibration after equilibration with USGS42 (human hair, non-exchangeable δ^2^H_V-SMOW_ = −44.4 ± 2.0‰), USGS43 (human hair, non-exchangeable δ^2^H_V-SMOW_ = −72.9 ± 2.2‰) and Eurofins 11/2/C (casein, non-exchangeable δ^2^H_V-SMOW_ = −113.37 ± 3.8‰) reference materials.

Dragonfly wings and exuviae, reference materials and laboratory standards were kept in the same room for at least three weeks before being wrapped in silver capsules. Sample weights ranged from 280 to 320 µg. Wrapped samples were kept in the analytical room for a further 5–6 days. Stable hydrogen isotope composition was determined using a Thermo Delta V Advantage continuous-flow IRMS (Thermo Scientific, Waltham, MA, USA) coupled with a Thermo Flash 2000 HT elemental analyzer via Conflo IV device. Samples were introduced using a Costech Zero Blank autosampler (Costech Analytical Technologies, Milan, Italy). The temperature of the glassy carbon reactor was set to 1420 °C. Reference materials or laboratory standards were analyzed before and after each run, as well as after every fifth unknown. The isotopic composition of H was expressed in a conventional δ-notation relative to the international standard (VSMOW). Analytical error (SD, n = 6) of the determination of isotopic composition in standard materials did not exceed 2‰.

### 2.3. Data Analysis

Data met assumptions of parametric analysis and were therefore treated using one-way ANOVA and Tukey’s HSD test for unequal sample sizes. Due to the lack of complete data in [7], δ^2^H values of *P. flavescens* caught in the Maldives were compared with other groups using the *t*-test (*p* < 0.05). Statistical analysis was performed in SPSS 16.0 and MS Excel. The mean and standard deviation are given to indicate the central tendency and variability. SimpleMappr, an online tool to produce publication-quality point maps, was used for making a schematic map of migrations [26].

## 3. Results

**Figure 2:**
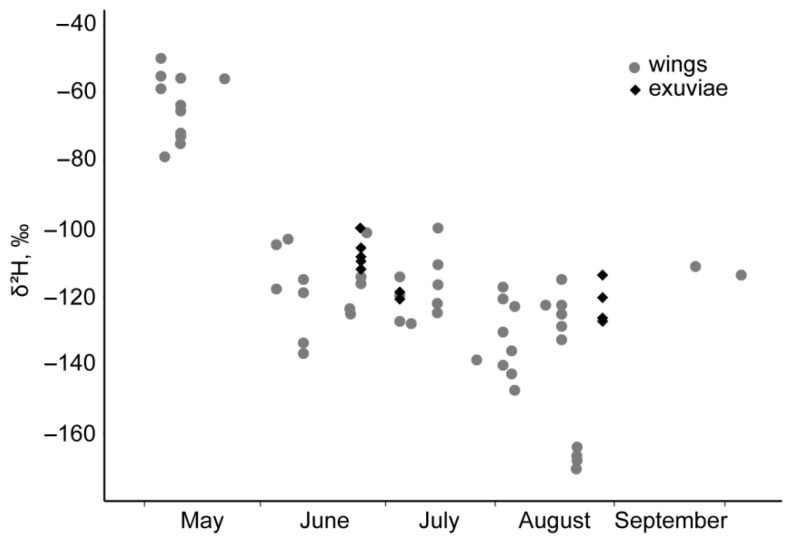
Changes in the δ^2^H values of the wings and exuviae of *Pantala flavescens* from Middle Asia depending on the date of collection.

The δ^2^H values of the wings and exuviae of *P. flavescens* from Middle Asia differed substantially depending on the time of collection (Figure 2). In the wings of specimens collected in May during oviposition, δ^2^H values ranged from −78.7 to −49.6‰, averaging −64.4 (SD 9.7)‰ (n = 12). Both the wings and exuviae of resident specimens collected from June to October had considerably lower δ^2^H values that varied from −170.9 to −91.7 ‰, and averaged −123.5 (17.2)‰ (n = 52).

**Figure 3:**
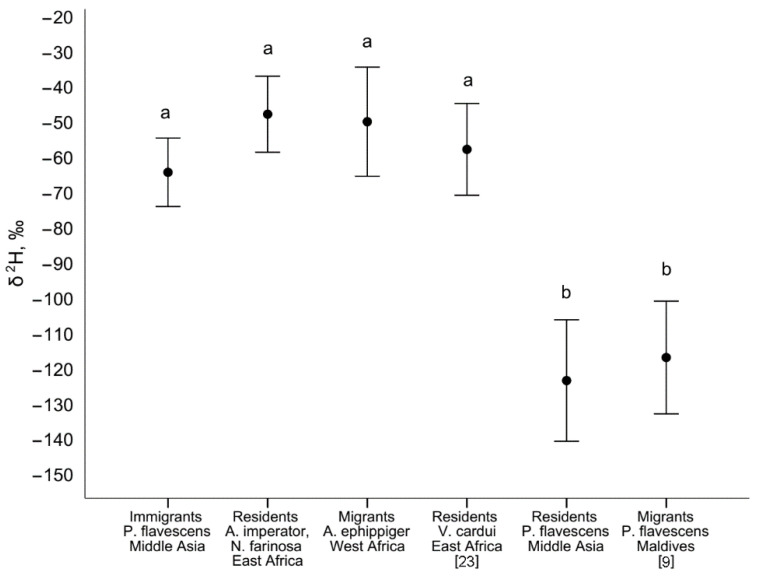
Comparison of the δ^2^H values of immigrants and residents of *Pantala flavescens* from Middle Asia with resident species of dragonflies (*Anax ephippiger*, *A. imperator*, *Nesciothemis farinosa*) and butterflies (*Vanessa cardui*) [23] from Africa, and *P. flavescens* during autumn migrations through the Maldives [9]. Significant differences between the groups are indicated by different letters (unequal n Tukey’s HSD, *p* < 0.05; to compare *P. flavescens* from the Maldives with other groups, a *t*-test was used, *p* < 0.05). Mean values and 1 SD are shown.

The δ^2^H values were similar in the wings of aboriginal dragonflies from Ethiopia (−47.9 (10.8)‰, n = 4, range from −60.7 to −34.5‰), *Anax ephippiger* from Mauritania (−50.1 (15.5)‰, n = 11, range from −76.8 to −16.8‰), and in the wings of *Vanessa cardui* butterflies from Ethiopia (−57.9 (13.0)‰, n = 21). These δ^2^H values did not differ from δ^2^H values in the wings of *P. flavescens* from Middle Asia collected in May (Figure 3). In contrast, δ^2^H values in the wings of *P. flavescens* collected in the Maldives (−117 (16)‰, n = 49) were similar to those in the wings and exuviae of *P. flavescens* from Middle Asia collected from June to October (Figure 3).

## 4. Discussion

### 4.1. P. flavescens in Middle Asia and Isotope Evidence of Spring Immigration

*P. flavescens* is found in Middle Asia only in the warm period of the year. It is highly unlikely that dragonflies can survive winter at preimaginal stages, as they require high temperatures. The minimum water temperature for egg development is 14.3 °C [27]. Larvae are not able to tolerate low temperatures or hibernate at high latitudes [5,28,29]. For instance, in Japan, larvae die at temperatures below 4 °C and are not able to hibernate even on the southern island of Honshu [5].

Imagoes of *P. flavescens* appear in Middle Asia in spring and are recorded in ponds during reproduction actions [11,30]. The high δ^2^H values of dragonflies collected during oviposition in May and their significant difference from the isotope signatures of local individuals (−64.4 (9.7)‰ and −123.5 (17.2)‰, respectively) indicate that they developed much farther south. This confirms the spring immigration of *P. flavescens* to Middle Asia.

To estimate the development time of the summer generation, we used the intensive breeding of dragonflies in rice paddies, their most common habitat in Middle Asia, although temperature allows some specimens to develop earlier (e.g., some *P. flavescens* immigrants in southwestern Tajikistan were recorded on April 26 [11]). Dragonflies begin to lay eggs on rice paddies at the time of water admission. Considering the dates of rice paddy water admission (first days of May) and the appearance of the first winged individuals of the summer generation, the approximate duration of preimaginal *P. flavescens* development in Tajikistan can be estimated as about two months [11]. This fits published data from North India (50–60days) [31] and South Australia (51 days) [32]. In other regions, the preimaginal development of *P. flavescens* can last from 30 to 65 days [8,33].

The total residence period of *P. flavescens* in Middle Asia is approximately six months. The latest single finds of these dragonflies were made in mid-October but most of these dragonflies, which are quite numerous in summer, “disappear” at the end of August. At this time, they migrate southward. Directional migrations were recorded in early August in the Alai Valley (3200 m a.s.l.) in Kyrgyzstan and in the Uy-bulak Pass (4260 m a.s.l.) in the Eastern Pamirs in Tajikistan [11].

### 4.2. The Probable Region of Origin of Immigrants Is East Africa and the Arabian Peninsula

Immigrant *P. flavescens* arrive in Middle Asia in spring from somewhere in the south. Their natal territory can be delimited using data on the phenology of this species in different regions. The arrival of *P. flavescens* from the Indian subcontinent in spring is excluded, since these dragonflies do not live in the north, west or most of the central regions of India in the winter, and arrive there for breeding with the south-western monsoon in June at the earliest [28,31,34,35,36]. In Middle Asia, as indicated above, the emergence of native dragonflies is already taking place at this time [11]. South-west Asia (except for the southern half of the Arabian Peninsula) can also be excluded from the potential natal area, since *P. flavescens* does not develop in the Palaearctic part of the range in winter. This is evidenced, for example, by numerous works on the dragonflies of Iran, which are summarized in the checklist [37]. Palaearctic Africa can also be excluded from the likely natal areas of *P. flavescens*. Finds of this species are rare here and are apparently associated with occasional migrations [38,39].

The development of *P. flavescens* in winter is expected in the tropical part of East Africa. Globe skimmers arrive in this region from October to December [7,8,9,40]. In the southern part of the Arabian Peninsula, *P. flavescens* is common from March to April and October to January, and develops in winter [41]. For example, in the United Arab Emirates and Oman, these dragonflies lay eggs from October to November [42], and in the south of Oman, emergence has been observed in March [43].

Thus, phenological data indicate a possible spring migration of *P. flavescens* to Middle Asia from the tropical parts of East Africa and the Arabian Peninsula. The similarity of the δ^2^H values of the wings of spring immigrant *P. flavescens* from Middle Asia and dragonflies that developed in Ethiopia and the Sahel region also strongly supports the hypothesis of African origin. The δ^2^H values of *Vanessa cardui* butterflies that developed in November in Ethiopia turned out to be close as well [23] (Figure 3). The δ^2^H values in the wings of dragonflies are associated with the δ^2^H values of atmospheric precipitation in the areas of insect origin, and this relationship is similar in different species of dragonflies, and also in other insects [14].

### 4.3. Probable Migration Pattern in the Afro-Asian Region

**Figure 4:**
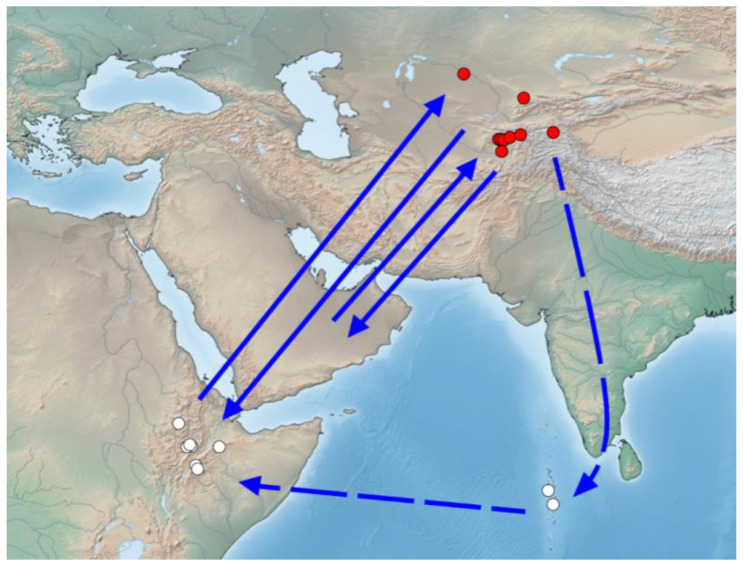
Supposed migrations of *Pantala flavescens* in the Afro-Asian region. Red dots indicate collection sites of *P. flavescens* in Middle Asia. White dots indicate collection sites of aboriginal dragonflies (sampled in this study) and butterflies [23] in East Africa, and collection sites of *P. flavescens* migrating through the Maldives archipelago [9].

The existence of a giant migration circuit of *P. flavescens* between Africa and India has been assumed since the discovery that these dragonflies fly from India to Africa across the Arabian Sea with a landing in the Maldives. It was supposed that such migrations occur over two to four generations [7]. Later, using the isotope method, it was suggested that dragonflies fly through the Maldives not from Central and South India, but from the northernmost part of the Hindustan Peninsula or even further north and east [9]. Our data show that the migration circuit of *P. flavescens* can be even larger. In spring, dragonflies migrate from Africa and the Arabian Peninsula to the northern edge of Middle Asia (Figure 1, locality 1, the lower reaches of the Syr Darya, Kyzylorda, 44°50′ N), covering a distance of 3000–5000 km. Their descendants, which develop near the northern limits of the range, migrate southward in autumn. Isotope signatures of dragonflies from Middle Asia and dragonflies that were caught during migrations in the Maldives are very similar (Figure 3). This suggests that at least some of the dragonflies can migrate from Middle Asia to Africa through the Maldives in autumn. In addition, these dragonflies make a long detour of more than 3500 km, crossing the Arabian Sea. In this case, the total length of migration route of *P. flavescens* could be more than 14,000 km (Figure 4). This includes migrations of two generations: winter African and Arabian, and summer Middle Asian.

Such large-scale migrations suggest the possibility of the prolonged existence of *P. flavescens* in the imaginal phase. The duration of the imaginal phase and puberty as well as the number of generations of this species are debated [5,28]. A misconception about the multivoltine development of *P. flavescens* in the tropical parts of the range can be formed, if there are several migration waves of dragonflies in the region, such as in South India [28]. Nevertheless, published data suggest that only one generation of *P. flavescens* develops in South India, and the development of the species is univoltine in North and West India [28,31,34,35]. In Middle Asia, it can be presumed that only one summer generation develops, because reproductive actions among dragonflies were not observed here in the second half of summer [11]. In Hong Kong, *P. flavescens* arrive with autumn typhoons in late September and October, and emergence occurs between November and January. It has also been shown that dragonflies can exist in the imaginal phase for several months [44]. A similar pattern is observed in South Australia [32]. It can be assumed that the lifespan of *P. flavescens* at adulthood is at least six months and most of this time is spent on migrations and vagrancy. Similarly, long imaginal life is common for *Anax ephippiger,* another well-known migratory species [20,21,22].

In regions adjacent to the Arabian Sea, air circulation has pronounced seasonal differences, known as monsoon circulation. The Arabian branch of the summer monsoon has a north-eastern direction here. It should be noted that in spring, when the monsoons change, the effect of the Iranian branch of the polar front is pronounced in South-west Asia. In spring (March to April), the Iranian branch of the polar front begins to displace to the north and passes through Middle Asia [45]. From February to April, northward winds prevail over East Africa and the Arabian Peninsula, while in the northern latitudes, a westerly current appears, carrying the air masses in a northeast direction to eastern Kazakhstan and beyond (Supplementary Figure S1a) [24]. At this time, spring migrations of *P. flavescens* take place to the north and north-east, including to Middle Asia. *P. flavescens* arrive in North and West India with the south-west monsoon much later, in June. The winter monsoon (or post-monsoon), on the contrary, moves air masses from the north-east to the south-west (Supplementary Figure S1b) and contributes to the autumn migrations of *P. flavescens* to the south-west (Figure 4).

In general, the migration routes of *P. flavescens* in the Afro-Asian region are still rather unclear. Apparently, the circular flight through the Maldives described above is only the maximum flyway during autumn migrations, which is common only for some of the dragonflies. Other individuals fly directly from Middle Asia to East Africa and the Arabian Peninsula. For instance, the appearance of large numbers of *P. flavescens* in the south-east of the Arabian Peninsula in October and November [42,46,47] is likely related to migration from more northerly territories, including Middle Asia. The duration and altitude of migratory dragonflies’ flights, specific routes and frequency of stops along the way are still a mystery, although there is some evidence of stepwise migrations over the ocean [7].

The adaptive significance of seasonal trans-latitudinal migrations of *P. flavescens* should be noted. It can be viewed as a mechanism of the fullest use of environmental resources in space and time, including the northern limits of the range where wintering is impossible. A successful reproduction of *P. flavescens* in Middle Asia illustrates the efficiency of this migration strategy. The number of individuals of the summer generation here is many times higher than that of their ancestors, which arrive in spring [11,48]. This seems to support the thesis that the ecological success of migratory insects depends largely on summer reproduction in high-latitude regions [49]. The significance of long-distance migrations of dragonflies as a part of the global phenomenon of insect mass migrations should be also emphasized, as the importance and scale of this phenomenon remain largely underestimated [50].

## 5. Conclusions

Stable isotope analysis of hydrogen in the wings of *P. flavescens* from Middle Asia was conducted for the first time. It showed a strong difference in δ^2^H values between the spring wave, apparently of immigrants, and the local generation of dragonflies. Phenological data on *P. flavescens* in the Afro-Asian region and a comparison with published isotopic data on migratory insects suggest that the probable area of origin of *P. flavescens* immigrants is located in tropical parts of East Africa and/or the Arabian Peninsula. Moreover, autumn migration of Middle Asian residents to the south may also pass through the Indian Ocean. We assume that in the Afro-Asian region there is an extensive migration circle of *P. flavescens* covering East Africa, Central Asia and the Indian subcontinent, with a total length of more than 14,000 km.

## Figures and Tables

**Table 1:** Localities and dates of collection of *Pantala flavescens* in Middle Asia (see Figure 1).

Locality	n	Wings/ Exuviae	Date of Collection	Country	Nearest Settlement	Altitude, m a.s.l.	Latitude, N	Longitude, E
1	1	wings	22.V.1976	Kazakhstan	Kyzylorda	170	44°50′	64°55′
1	5	wings	18.VIII.1976	Kazakhstan	Kyzylorda	170	44°50′	64°55′
2	1	wings	14.VIII.1973	Kazakhstan	Chokpak	1183	42°31′	70°36′
2	1	wings	04.X.2010	Kazakhstan	Chokpak	1183	42°31′	70°36′
3	2	wings	05.VI.1978	Tajikistan	Tigrovaya Balka	340	37°25′	68°30′
3	1	wings	08.VI.1978	Tajikistan	Tigrovaya Balka	340	37°25′	68°30′
3	4	wings	12.VI.1979	Tajikistan	Tigrovaya Balka	340	37°25′	68°30′
3	2	wings	24.VI.1978	Tajikistan	Tigrovaya Balka	340	37°25′	68°30′
3	1	wings	28.VI.1982	Tajikistan	Tigrovaya Balka	340	37°25′	68°30′
3	1	wings	10.VII.1984	Tajikistan	Tigrovaya Balka	340	37°25′	68°30′
3	1	wings	27.VII.1977	Tajikistan	Tigrovaya Balka	340	37°25′	68°30′
3	2	wings	05.VIII.1978	Tajikistan	Tigrovaya Balka	340	37°25′	68°30′
3	2	wings	06.VIII.1979	Tajikistan	Tigrovaya Balka	340	37°25′	68°30′
4	3	wings	06.V.2019	Tajikistan	Regar	732	38°32′	68°13′
4	1	wings	07.V.2019	Tajikistan	Regar	732	38°32′	68°13′
4	7	wings	11.V.2019	Tajikistan	Regar	732	38°32′	68°13′
5	5	wings	17.VII.1988	Tajikistan	Shakhrinau	850	38°34′	68°20′
6	3	wings	07.VII.1988	Tajikistan	Gissar	712	38°28′	68°36′
6	2	exuviae	07.VII.1988	Tajikistan	Gissar	712	38°28′	68°36′
6	1	wings	22.IX.1991	Tajikistan	Gissar	712	38°28′	68°36′
7	2	wings	27.VI.1981	Tajikistan	Ramit	1195	38°44′	69°19′
7	5	exuviae	27.VI.1981	Tajikistan	Ramit	1195	38°44′	69°19′
8	4	wings	22.VIII.1987	Tajikistan	Garm	1300	39°00′	70°18′
8	4	exuviae	29.VIII.1987	Tajikistan	Garm	1300	39°00′	70°18′
9	4	wings	03.VIII.1980	Tajikistan	Uy-bulak pass ^1^	4260	39°12′	73°26′

^1^ Dragonflies were caught during autumn migrations in the high mountains of the Eastern Pamirs at Uy-bulak Pass (Tajikistan).

## Supplementary Materials

The following are available online at https://www.mdpi.com/2075-4450/11/12/890/s1, Table S1: Hydrogen isotope composition (δ^2^H values) of wings and exuviae of *Pantala flavescens* in Middle Asia, Figure S1: Mean wind vectors at 850 mb in Afro-Asian region during proposed migrations of *Pantala flavescens* (a) to the north in early spring (February to April) and (b) to the south in autumn (September to November). Images are provided by the NOAA/ESRL Physical Sciences Laboratory, Boulder Colorado, http://psl.noaa.gov/, data for 1976–2000 [24].
