# Supplementary material for: Seasonal Migrations of Pantala flavescens (Odonata: Libellulidae) in Middle Asia and Understanding of the Migration Model in the Afro-Asian Region Using Stable Isotopes of Hydrogen
Source: Insects. 2020 Dec 17;11(12):890. doi: 10.3390/insects11120890 (PMC7765977; doi:10.3390/insects11120890)

**Supplementary Figure S1.** Mean wind vectors at 850 mb in Afro-Asian region during proposed migrations of *Pantala flavescens* (a) to the north in early spring (February to April) and (b) to the south in autumn (September to November). Images are provided by the NOAA/ESRL Physical Sciences Laboratory, Boulder Colorado, http://psl.noaa.gov/, data for 1976-2000 [24].


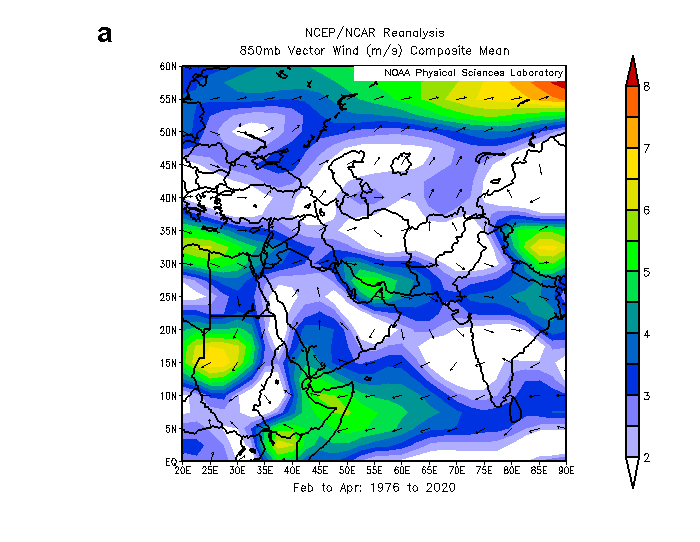


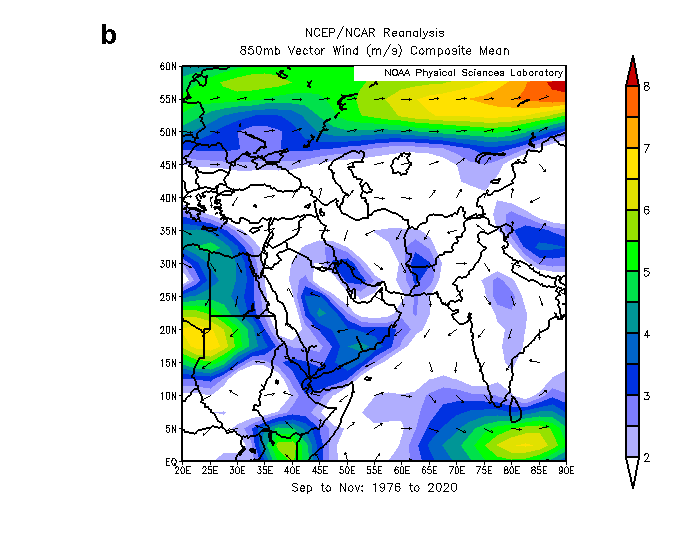

Supplement: Supplementary file 1 [file insects-11-00890-s001.zip › Supplementary_Figure_S1.docx]
